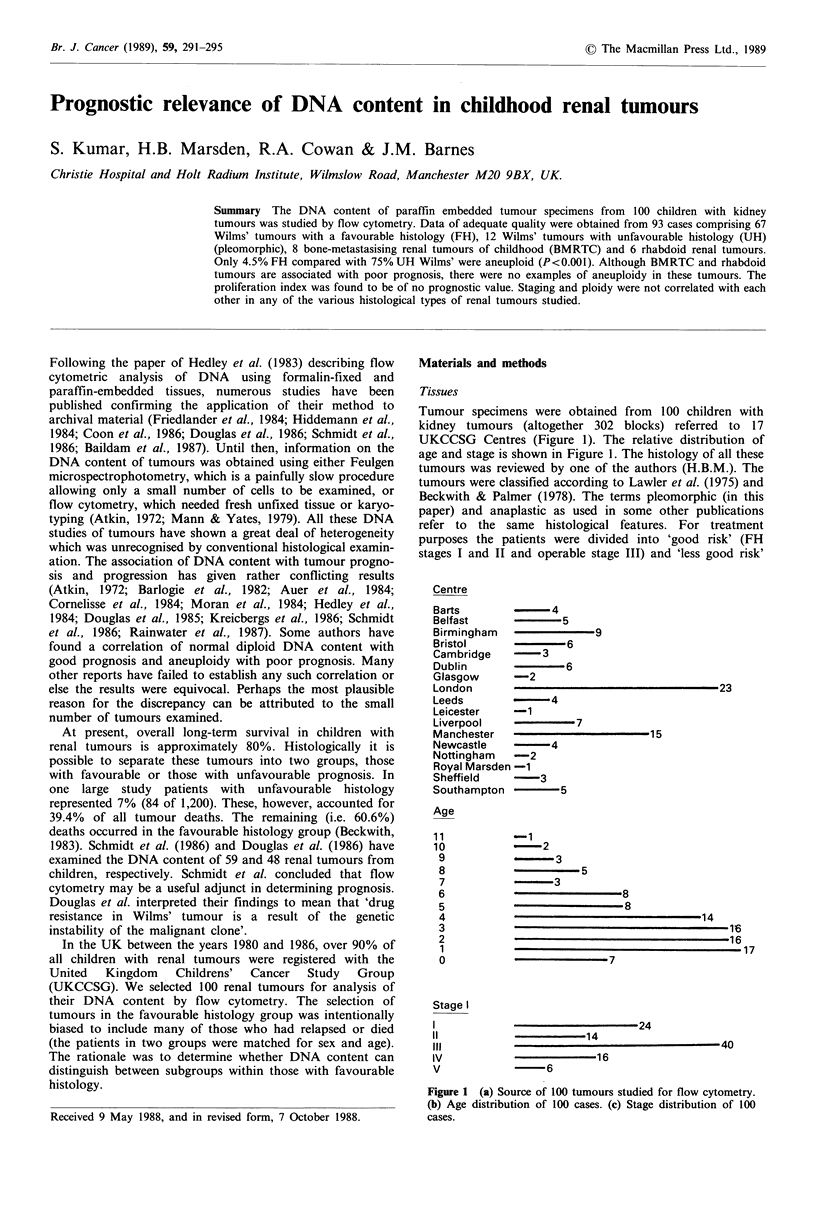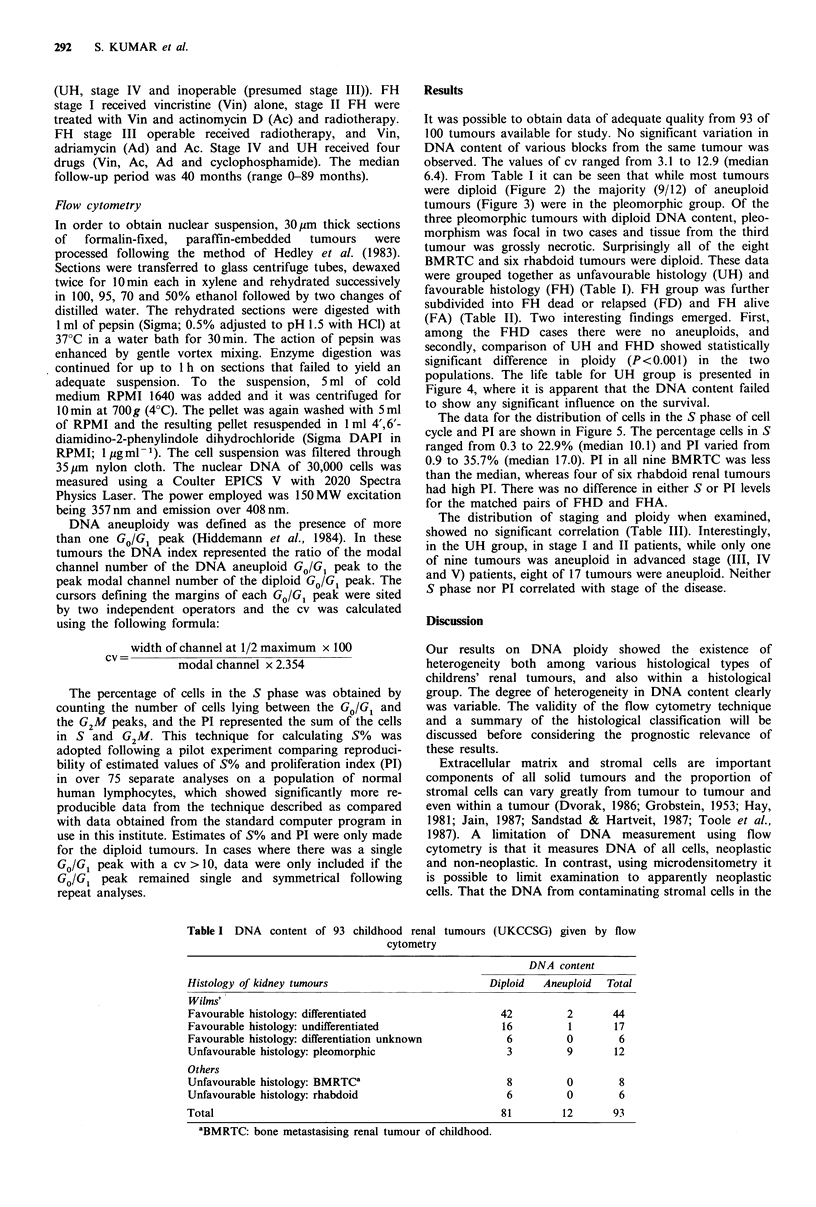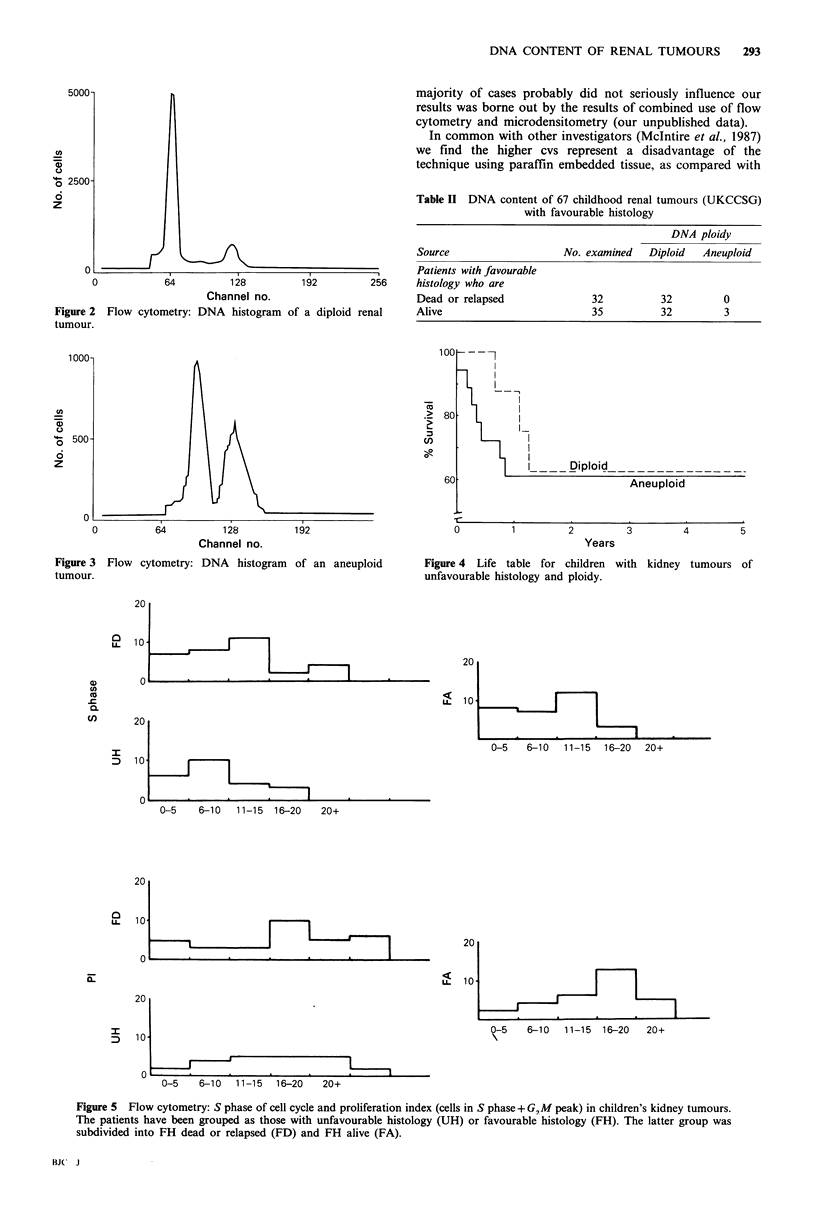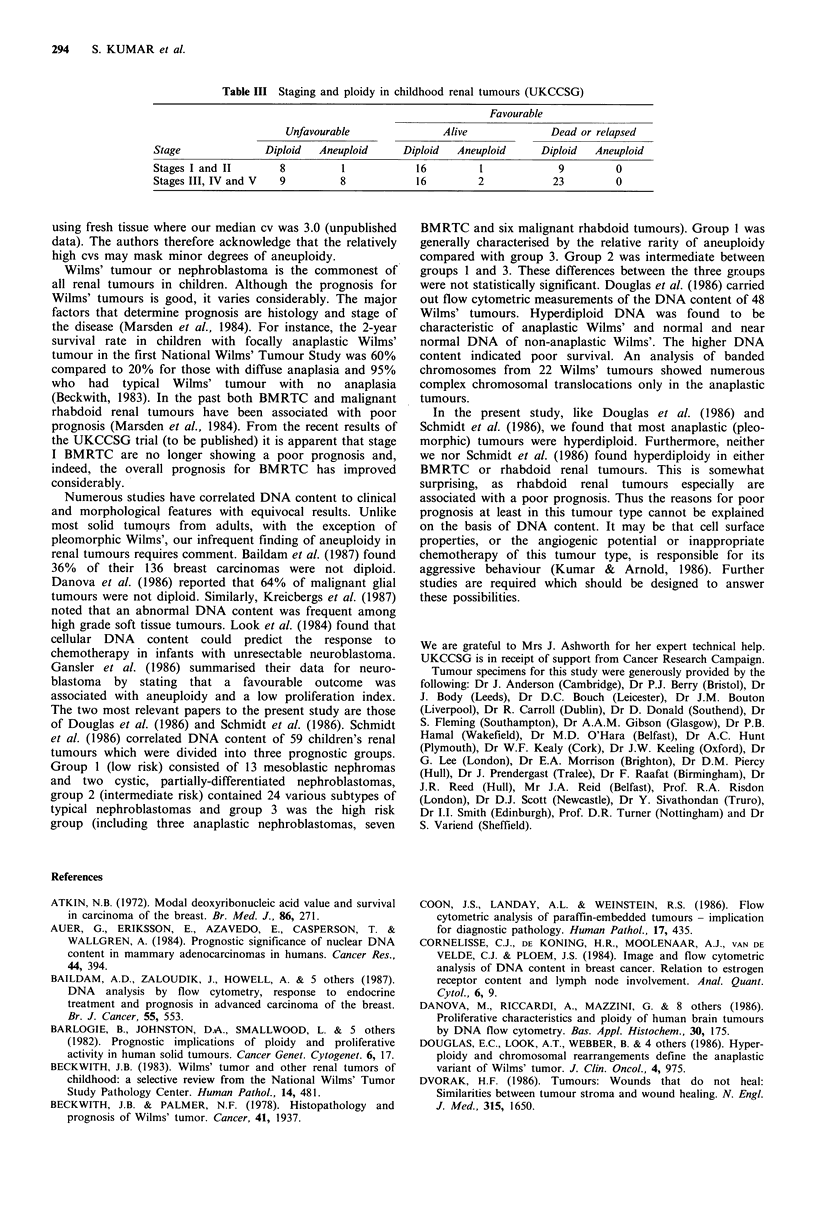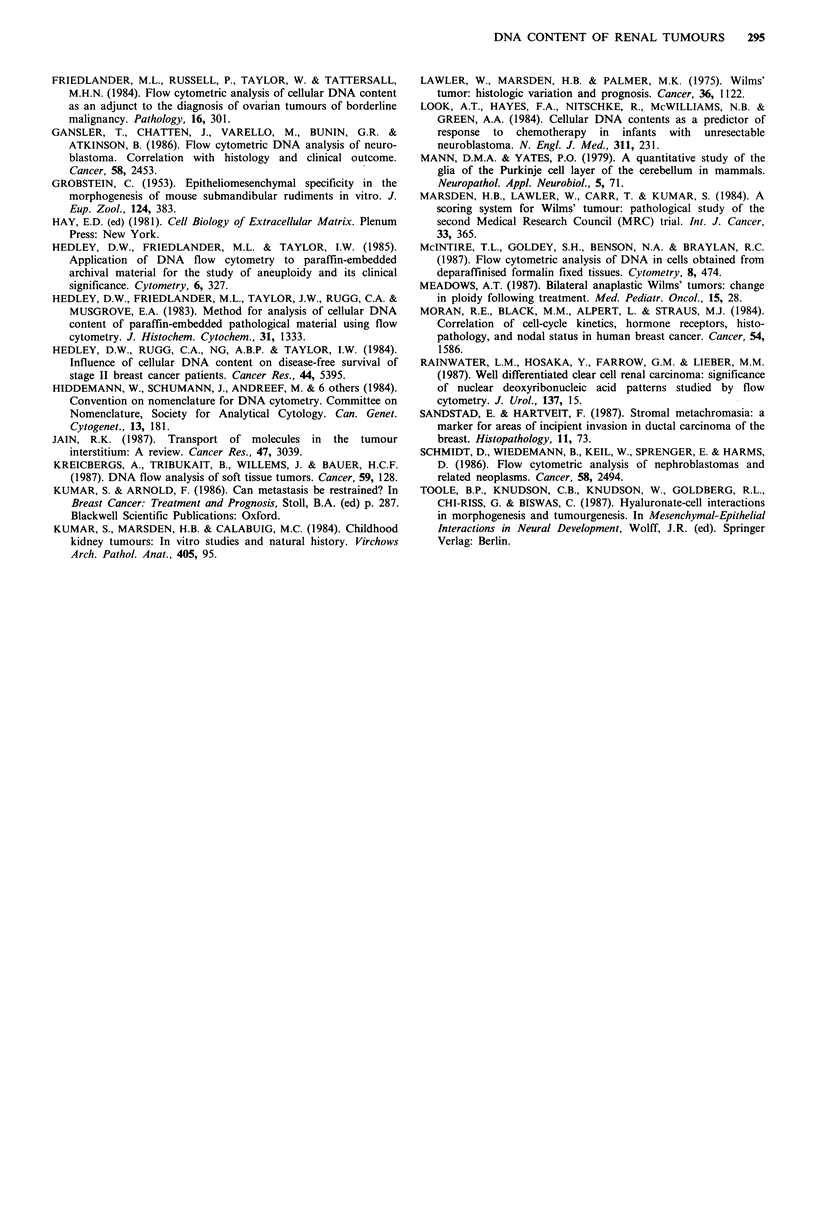# Prognostic relevance of DNA content in childhood renal tumours.

**DOI:** 10.1038/bjc.1989.60

**Published:** 1989-02

**Authors:** S. Kumar, H. B. Marsden, R. A. Cowan, J. M. Barnes

**Affiliations:** Christie Hospital, Holt Radium Institute, Manchester, UK.

## Abstract

The DNA content of paraffin embedded tumour specimens from 100 children with kidney tumours was studied by flow cytometry. Data of adequate quality were obtained from 93 cases comprising 67 Wilms' tumours with a favourable histology (FH), 12 Wilms' tumours with unfavourable histology (UH) (pleomorphic), 8 bone-metastasising renal tumours of childhood (BMRTC) and 6 rhabdoid renal tumours. Only 4.5% FH compared with 75% UH Wilms' were aneuploid (P less than 0.001). Although BMRTC and rhabdoid tumours are associated with poor prognosis, there were no examples of aneuploidy in these tumours. The proliferation index was found to be of no prognostic value. Staging and ploidy were not correlated with each other in any of the various histological types of renal tumours studied.


*Br. J. Cancer* (1989), **59**, 291–295 © The Macmillan Press Ltd., 1989

### Prognostic relevance of DNA content in childhood renal tumours

S. Kumar, H.B. Marsden, R.A. Cowan & J.M. Barnes

*Christie Hospital and Holt Radium Institute, Wilmslow Road, Manchester M20 9BX, UK.*

**Summary** The DNA content of paraffin embedded tumour specimens from 100 children with kidney tumours was studied by flow cytometry. Data of adequate quality were obtained from 93 cases comprising 67 Wilms' tumours with a favourable histology (FH), 12 Wilms' tumours with unfavourable histology (UH) (pleomorphic), 8 bone-metastasising renal tumours of childhood (BMRTC) and 6 rhabdoid renal tumours. Only 4.5% FH compared with 75% UH Wilms' were aneuploid ($P<0.001$). Although BMRTC and rhabdoid tumours are associated with poor prognosis, there were no examples of aneuploidy in these tumours. The proliferation index was found to be of no prognostic value. Staging and ploidy were not correlated with each other in any of the various histological types of renal tumours studied.

Following the paper of Hedley *et al.* (1983) describing flow cytometric analysis of DNA using formalin-fixed and paraffin-embedded tissues, numerous studies have been published confirming the application of their method to archival material (Friedlander *et al.*, 1984; Hiddemann *et al.*, 1984; Coon *et al.*, 1986; Douglas *et al.*, 1986; Schmidt *et al.*, 1986; Baildam *et al.*, 1987). Until then, information on the DNA content of tumours was obtained using either Feulgen microspectrophotometry, which is a painfully slow procedure allowing only a small number of cells to be examined, or flow cytometry, which needed fresh unfixed tissue or karyotyping (Atkin, 1972; Mann & Yates, 1979). All these DNA studies of tumours have shown a great deal of heterogeneity which was unrecognised by conventional histological examination. The association of DNA content with tumour prognosis and progression has given rather conflicting results (Atkin, 1972; Barlogie *et al.*, 1982; Auer *et al.*, 1984; Cornelisse *et al.*, 1984; Moran *et al.*, 1984; Hedley *et al.*, 1984; Douglas *et al.*, 1985; Kreicbergs *et al.*, 1986; Schmidt *et al.*, 1986; Rainwater *et al.*, 1987). Some authors have found a correlation of normal diploid DNA content with good prognosis and aneuploidy with poor prognosis. Many other reports have failed to establish any such correlation or else the results were equivocal. Perhaps the most plausible reason for the discrepancy can be attributed to the small number of tumours examined.

At present, overall long-term survival in children with renal tumours is approximately 80%. Histologically it is possible to separate these tumours into two groups, those with favourable or those with unfavourable prognosis. In one large study patients with unfavourable histology represented 7% (84 of 1,200). These, however, accounted for 39.4% of all tumour deaths. The remaining (i.e. 60.6%) deaths occurred in the favourable histology group (Beckwith, 1983). Schmidt *et al.* (1986) and Douglas *et al.* (1986) have examined the DNA content of 59 and 48 renal tumours from children, respectively. Schmidt *et al.* concluded that flow cytometry may be a useful adjunct in determining prognosis. Douglas *et al.* interpreted their findings to mean that 'drug resistance in Wilms' tumour is a result of the genetic instability of the malignant clone'.

In the UK between the years 1980 and 1986, over 90% of all children with renal tumours were registered with the United Kingdom Childrens' Cancer Study Group (UKCCSG). We selected 100 renal tumours for analysis of their DNA content by flow cytometry. The selection of tumours in the favourable histology group was intentionally biased to include many of those who had relapsed or died (the patients in two groups were matched for sex and age). The rationale was to determine whether DNA content can distinguish between subgroups within those with favourable histology.

Received 9 May 1988, and in revised form, 7 October 1988.

### Materials and methods

### *Tissues*

Tumour specimens were obtained from 100 children with kidney tumours (altogether 302 blocks) referred to 17 UKCCSG Centres (Figure 1). The relative distribution of age and stage is shown in Figure 1. The histology of all these tumours was reviewed by one of the authors (H.B.M.). The tumours were classified according to Lawler *et al.* (1975) and Beckwith & Palmer (1978). The terms pleomorphic (in this paper) and anaplastic as used in some other publications refer to the same histological features. For treatment purposes the patients were divided into 'good risk' (FH stages I and II and operable stage III) and 'less good risk'

**Figure 1** (**a**) Source of 100 tumours studied for flow cytometry. (**b**) Age distribution of 100 cases. (**c**) Stage distribution of 100 cases.

(UH, stage IV and inoperable (presumed stage III)). FH stage I received vincristine (Vin) alone, stage II FH were treated with Vin and actinomycin D (Ac) and radiotherapy. FH stage III operable received radiotherapy, and Vin, adriamycin (Ad) and Ac. Stage IV and UH received four drugs (Vin, Ac, Ad and cyclophosphamide). The median follow-up period was 40 months (range 0–89 months).

*Flow cytometry*

In order to obtain nuclear suspension, 30 $\mu$m thick sections of formalin-fixed, paraffin-embedded tumours were processed following the method of Hedley *et al.* (1983). Sections were transferred to glass centrifuge tubes, dewaxed twice for 10 min each in xylene and rehydrated successively in 100, 95, 70 and 50% ethanol followed by two changes of distilled water. The rehydrated sections were digested with 1 ml of pepsin (Sigma; 0.5% adjusted to pH 1.5 with HCl) at 37°C in a water bath for 30 min. The action of pepsin was enhanced by gentle vortex mixing. Enzyme digestion was continued for up to 1 h on sections that failed to yield an adequate suspension. To the suspension, 5 ml of cold medium RPMI 1640 was added and it was centrifuged for 10 min at 700 *g* (4°C). The pellet was again washed with 5 ml of RPMI and the resulting pellet resuspended in 1 ml 4′,6′-diamidino-2-phenylindole dihydrochloride (Sigma DAPI in RPMI; 1 $\mu$g ml$^{-1}$). The cell suspension was filtered through 35 $\mu$m nylon cloth. The nuclear DNA of 30,000 cells was measured using a Coulter EPICS V with 2020 Spectra Physics Laser. The power employed was 150 MW excitation being 357 nm and emission over 408 nm.

DNA aneuploidy was defined as the presence of more than one $G_0/G_1$ peak (Hiddemann *et al.*, 1984). In these tumours the DNA index represented the ratio of the modal channel number of the DNA aneuploid $G_0/G_1$ peak to the peak modal channel number of the diploid $G_0/G_1$ peak. The cursors defining the margins of each $G_0/G_1$ peak were sited by two independent operators and the cv was calculated using the following formula:

$$\text{cv} = \frac{\text{width of channel at 1/2 maximum} \times 100}{\text{modal channel} \times 2.354}$$

The percentage of cells in the *S* phase was obtained by counting the number of cells lying between the $G_0/G_1$ and the $G_2M$ peaks, and the PI represented the sum of the cells in *S* and $G_2M$. This technique for calculating *S*% was adopted following a pilot experiment comparing reproducibility of estimated values of *S*% and proliferation index (PI) in over 75 separate analyses on a population of normal human lymphocytes, which showed significantly more reproducible data from the technique described as compared with data obtained from the standard computer program in use in this institute. Estimates of *S*% and PI were only made for the diploid tumours. In cases where there was a single $G_0/G_1$ peak with a cv > 10, data were only included if the $G_0/G_1$ peak remained single and symmetrical following repeat analyses.

### Results

It was possible to obtain data of adequate quality from 93 of 100 tumours available for study. No significant variation in DNA content of various blocks from the same tumour was observed. The values of cv ranged from 3.1 to 12.9 (median 6.4). From Table I it can be seen that while most tumours were diploid (Figure 2) the majority (9/12) of aneuploid tumours (Figure 3) were in the pleomorphic group. Of the three pleomorphic tumours with diploid DNA content, pleomorphism was focal in two cases and tissue from the third tumour was grossly necrotic. Surprisingly all of the eight BMRTC and six rhabdoid tumours were diploid. These data were grouped together as unfavourable histology (UH) and favourable histology (FH) (Table I). FH group was further subdivided into FH dead or relapsed (FD) and FH alive (FA) (Table II). Two interesting findings emerged. First, among the FHD cases there were no aneuploids, and secondly, comparison of UH and FHD showed statistically significant difference in ploidy ($P$<0.001) in the two populations. The life table for UH group is presented in Figure 4, where it is apparent that the DNA content failed to show any significant influence on the survival.

The data for the distribution of cells in the *S* phase of cell cycle and PI are shown in Figure 5. The percentage cells in *S* ranged from 0.3 to 22.9% (median 10.1) and PI varied from 0.9 to 35.7% (median 17.0). PI in all nine BMRTC was less than the median, whereas four of six rhabdoid renal tumours had high PI. There was no difference in either *S* or PI levels for the matched pairs of FHD and FHA.

The distribution of staging and ploidy when examined, showed no significant correlation (Table III). Interestingly, in the UH group, in stage I and II patients, while only one of nine tumours was aneuploid in advanced stage (III, IV and V) patients, eight of 17 tumours were aneuploid. Neither *S* phase nor PI correlated with stage of the disease.

### Discussion

Our results on DNA ploidy showed the existence of heterogeneity both among various histological types of childrens' renal tumours, and also within a histological group. The degree of heterogeneity in DNA content clearly was variable. The validity of the flow cytometry technique and a summary of the histological classification will be discussed before considering the prognostic relevance of these results.

Extracellular matrix and stromal cells are important components of all solid tumours and the proportion of stromal cells can vary greatly from tumour to tumour and even within a tumour (Dvorak, 1986; Grobstein, 1953; Hay, 1981; Jain, 1987; Sandstad & Hartveit, 1987; Toole *et al.*, 1987). A limitation of DNA measurement using flow cytometry is that it measures DNA of all cells, neoplastic and non-neoplastic. In contrast, using microdensitometry it is possible to limit examination to apparently neoplastic cells. That the DNA from contaminating stromal cells in the

**Table I** DNA content of 93 childhood renal tumours (UKCCSG) given by flow cytometry

| *Histology of kidney tumours* | *DNA content* Diploid | Aneuploid | Total |
|---|---|---|---|
| *Wilms'* | | | |
| Favourable histology: differentiated | 42 | 2 | 44 |
| Favourable histology: undifferentiated | 16 | 1 | 17 |
| Favourable histology: differentiation unknown | 6 | 0 | 6 |
| Unfavourable histology: pleomorphic | 3 | 9 | 12 |
| *Others* | | | |
| Unfavourable histology: BMRTC[a] | 8 | 0 | 8 |
| Unfavourable histology: rhabdoid | 6 | 0 | 6 |
| Total | 81 | 12 | 93 |

[a]BMRTC: bone metastasising renal tumour of childhood.

**Figure 2** Flow cytometry: DNA histogram of a diploid renal tumour.

**Figure 3** Flow cytometry: DNA histogram of an aneuploid tumour.

majority of cases probably did not seriously influence our results was borne out by the results of combined use of flow cytometry and microdensitometry (our unpublished data).

In common with other investigators (McIntire *et al.*, 1987) we find the higher cvs represent a disadvantage of the technique using paraffin embedded tissue, as compared with

**Table II** DNA content of 67 childhood renal tumours (UKCCSG) with favourable histology

| Source | No. examined | *DNA ploidy* | |
|---|---|---|---|
| | | *Diploid* | *Aneuploid* |
| *Patients with favourable histology who are* | | | |
| Dead or relapsed | 32 | 32 | 0 |
| Alive | 35 | 32 | 3 |

**Figure 4** Life table for children with kidney tumours of unfavourable histology and ploidy.

**Figure 5** Flow cytometry: *S* phase of cell cycle and proliferation index (cells in *S* phase + $G_2M$ peak) in children's kidney tumours. The patients have been grouped as those with unfavourable histology (UH) or favourable histology (FH). The latter group was subdivided into FH dead or relapsed (FD) and FH alive (FA).

**Table III** Staging and ploidy in childhood renal tumours (UKCCSG)

| | *Unfavourable* | | *Favourable* | | | |
|---|---|---|---|---|---|---|
| | | | *Alive* | | *Dead or relapsed* | |
| *Stage* | *Diploid* | *Aneuploid* | *Diploid* | *Aneuploid* | *Diploid* | *Aneuploid* |
| Stages I and II | 8 | 1 | 16 | 1 | 9 | 0 |
| Stages III, IV and V | 9 | 8 | 16 | 2 | 23 | 0 |

using fresh tissue where our median cv was 3.0 (unpublished data). The authors therefore acknowledge that the relatively high cvs may mask minor degrees of aneuploidy.

Wilms' tumour or nephroblastoma is the commonest of all renal tumours in children. Although the prognosis for Wilms' tumours is good, it varies considerably. The major factors that determine prognosis are histology and stage of the disease (Marsden *et al.*, 1984). For instance, the 2-year survival rate in children with focally anaplastic Wilms' tumour in the first National Wilms' Tumour Study was 60% compared to 20% for those with diffuse anaplasia and 95% who had typical Wilms' tumour with no anaplasia (Beckwith, 1983). In the past both BMRTC and malignant rhabdoid renal tumours have been associated with poor prognosis (Marsden *et al.*, 1984). From the recent results of the UKCCSG trial (to be published) it is apparent that stage I BMRTC are no longer showing a poor prognosis and, indeed, the overall prognosis for BMRTC has improved considerably.

Numerous studies have correlated DNA content to clinical and morphological features with equivocal results. Unlike most solid tumours from adults, with the exception of pleomorphic Wilms', our infrequent finding of aneuploidy in renal tumours requires comment. Baildam *et al.* (1987) found 36% of their 136 breast carcinomas were not diploid. Danova *et al.* (1986) reported that 64% of malignant glial tumours were not diploid. Similarly, Kreicbergs *et al.* (1987) noted that an abnormal DNA content was frequent among high grade soft tissue tumours. Look *et al.* (1984) found that cellular DNA content could predict the response to chemotherapy in infants with unresectable neuroblastoma. Gansler *et al.* (1986) summarised their data for neuroblastoma by stating that a favourable outcome was associated with aneuploidy and a low proliferation index. The two most relevant papers to the present study are those of Douglas *et al.* (1986) and Schmidt *et al.* (1986). Schmidt *et al.* (1986) correlated DNA content of 59 children's renal tumours which were divided into three prognostic groups. Group 1 (low risk) consisted of 13 mesoblastic nephromas and two cystic, partially-differentiated nephroblastomas, group 2 (intermediate risk) contained 24 various subtypes of typical nephroblastomas and group 3 was the high risk group (including three anaplastic nephroblastomas, seven BMRTC and six malignant rhabdoid tumours). Group 1 was generally characterised by the relative rarity of aneuploidy compared with group 3. Group 2 was intermediate between groups 1 and 3. These differences between the three groups were not statistically significant. Douglas *et al.* (1986) carried out flow cytometric measurements of the DNA content of 48 Wilms' tumours. Hyperdiploid DNA was found to be characteristic of anaplastic Wilms' and normal and near normal DNA of non-anaplastic Wilms'. The higher DNA content indicated poor survival. An analysis of banded chromosomes from 22 Wilms' tumours showed numerous complex chromosomal translocations only in the anaplastic tumours.

In the present study, like Douglas *et al.* (1986) and Schmidt *et al.* (1986), we found that most anaplastic (pleomorphic) tumours were hyperdiploid. Furthermore, neither we nor Schmidt *et al.* (1986) found hyperdiploidy in either BMRTC or rhabdoid renal tumours. This is somewhat surprising, as rhabdoid renal tumours especially are associated with a poor prognosis. Thus the reasons for poor prognosis at least in this tumour type cannot be explained on the basis of DNA content. It may be that cell surface properties, or the angiogenic potential or inappropriate chemotherapy of this tumour type, is responsible for its aggressive behaviour (Kumar & Arnold, 1986). Further studies are required which should be designed to answer these possibilities.

We are grateful to Mrs J. Ashworth for her expert technical help. UKCCSG is in receipt of support from Cancer Research Campaign.

Tumour specimens for this study were generously provided by the following: Dr J. Anderson (Cambridge), Dr P.J. Berry (Bristol), Dr J. Body (Leeds), Dr D.C. Bouch (Leicester), Dr J.M. Bouton (Liverpool), Dr R. Carroll (Dublin), Dr D. Donald (Southend), Dr S. Fleming (Southampton), Dr A.A.M. Gibson (Glasgow), Dr P.B. Hamal (Wakefield), Dr M.D. O'Hara (Belfast), Dr A.C. Hunt (Plymouth), Dr W.F. Kealy (Cork), Dr J.W. Keeling (Oxford), Dr G. Lee (London), Dr E.A. Morrison (Brighton), Dr D.M. Piercy (Hull), Dr J. Prendergast (Tralee), Dr F. Raafat (Birmingham), Dr J.R. Reed (Hull), Mr J.A. Reid (Belfast), Prof. R.A. Risdon (London), Dr D.J. Scott (Newcastle), Dr Y. Sivathondan (Truro), Dr I.I. Smith (Edinburgh), Prof. D.R. Turner (Nottingham) and Dr S. Variend (Sheffield).

### References

ATKIN, N.B. (1972). Modal deoxyribonucleic acid value and survival in carcinoma of the breast. *Br. Med. J.*, **86**, 271.

AUER, G., ERIKSSON, E., AZAVEDO, E., CASPERSON, T. & WALLGREN, A. (1984). Prognostic significance of nuclear DNA content in mammary adenocarcinomas in humans. *Cancer Res.*, **44**, 394.

BAILDAM, A.D., ZALOUDIK, J., HOWELL, A. & 5 others (1987). DNA analysis by flow cytometry, response to endocrine treatment and prognosis in advanced carcinoma of the breast. *Br. J. Cancer*, **55**, 553.

BARLOGIE, B., JOHNSTON, D.A., SMALLWOOD, L. & 5 others (1982). Prognostic implications of ploidy and proliferative activity in human solid tumours. *Cancer Genet. Cytogenet.* **6**, 17.

BECKWITH, J.B. (1983). Wilms' tumor and other renal tumors of childhood: a selective review from the National Wilms' Tumor Study Pathology Center. *Human Pathol.*, **14**, 481.

BECKWITH, J.B. & PALMER, N.F. (1978). Histopathology and prognosis of Wilms' tumor. *Cancer*, **41**, 1937.

COON, J.S., LANDAY, A.L. & WEINSTEIN, R.S. (1986). Flow cytometric analysis of paraffin-embedded tumours – implication for diagnostic pathology. *Human Pathol.*, **17**, 435.

CORNELISSE, C.J., DE KONING, H.R., MOOLENAAR, A.J., VAN DE VELDE, C.J. & PLOEM, J.S. (1984). Image and flow cytometric analysis of DNA content in breast cancer. Relation to estrogen receptor content and lymph node involvement. *Anal. Quant. Cytol.*, **6**, 9.

DANOVA, M., RICCARDI, A., MAZZINI, G. & 8 others (1986). Proliferative characteristics and ploidy of human brain tumours by DNA flow cytometry. *Bas. Appl. Histochem.*, **30**, 175.

DOUGLAS, E.C., LOOK, A.T., WEBBER, B. & 4 others (1986). Hyperploidy and chromosomal rearrangements define the anaplastic variant of Wilms' tumor. *J. Clin. Oncol.*, **4**, 975.

DVORAK, H.F. (1986). Tumours: Wounds that do not heal: Similarities between tumour stroma and wound healing. *N. Engl. J. Med.*, **315**, 1650.

FRIEDLANDER, M.L., RUSSELL, P., TAYLOR, W. & TATTERSALL, M.H.N. (1984). Flow cytometric analysis of cellular DNA content as an adjunct to the diagnosis of ovarian tumours of borderline malignancy. *Pathology*, **16**, 301.

GANSLER, T., CHATTEN, J., VARELLO, M., BUNIN, G.R. & ATKINSON, B. (1986). Flow cytometric DNA analysis of neuroblastoma. Correlation with histology and clinical outcome. *Cancer*, **58**, 2453.

GROBSTEIN, C. (1953). Epitheliomesenchymal specificity in the morphogenesis of mouse submandibular rudiments in vitro. *J. Eup. Zool.*, **124**, 383.

HAY, E.D. (ed) (1981). *Cell Biology of Extracellular Matrix*. Plenum Press: New York.

HEDLEY, D.W., FRIEDLANDER, M.L. & TAYLOR, I.W. (1985). Application of DNA flow cytometry to paraffin-embedded archival material for the study of aneuploidy and its clinical significance. *Cytometry*, **6**, 327.

HEDLEY, D.W., FRIEDLANDER, M.L., TAYLOR, J.W., RUGG, C.A. & MUSGROVE, E.A. (1983). Method for analysis of cellular DNA content of paraffin-embedded pathological material using flow cytometry. *J. Histochem. Cytochem.*, **31**, 1333.

HEDLEY, D.W., RUGG, C.A., NG, A.B.P. & TAYLOR, I.W. (1984). Influence of cellular DNA content on disease-free survival of stage II breast cancer patients. *Cancer Res.*, **44**, 5395.

HIDDEMANN, W., SCHUMANN, J., ANDREEF, M. & 6 others (1984). Convention on nomenclature for DNA cytometry. Committee on Nomenclature, Society for Analytical Cytology. *Can. Genet. Cytogenet.*, **13**, 181.

JAIN, R.K. (1987). Transport of molecules in the tumour interstitium: A review. *Cancer Res.*, **47**, 3039.

KREICBERGS, A., TRIBUKAIT, B., WILLEMS, J. & BAUER, H.C.F. (1987). DNA flow analysis of soft tissue tumors. *Cancer*, **59**, 128.

KUMAR, S. & ARNOLD, F. (1986). Can metastasis be restrained? In *Breast Cancer: Treatment and Prognosis*, Stoll, B.A. (ed) p. 287. Blackwell Scientific Publications: Oxford.

KUMAR, S., MARSDEN, H.B. & CALABUIG, M.C. (1984). Childhood kidney tumours: In vitro studies and natural history. *Virchows Arch. Pathol. Anat.*, **405**, 95.

LAWLER, W., MARSDEN, H.B. & PALMER, M.K. (1975). Wilms' tumor: histologic variation and prognosis. *Cancer*, **36**, 1122.

LOOK, A.T., HAYES, F.A., NITSCHKE, R., McWILLIAMS, N.B. & GREEN, A.A. (1984). Cellular DNA contents as a predictor of response to chemotherapy in infants with unresectable neuroblastoma. *N. Engl. J. Med.*, **311**, 231.

MANN, D.M.A. & YATES, P.O. (1979). A quantitative study of the glia of the Purkinje cell layer of the cerebellum in mammals. *Neuropathol. Appl. Neurobiol.*, **5**, 71.

MARSDEN, H.B., LAWLER, W., CARR, T. & KUMAR, S. (1984). A scoring system for Wilms' tumour: pathological study of the second Medical Research Council (MRC) trial. *Int. J. Cancer*, **33**, 365.

McINTIRE, T.L., GOLDEY, S.H., BENSON, N.A. & BRAYLAN, R.C. (1987). Flow cytometric analysis of DNA in cells obtained from deparaffinised formalin fixed tissues. *Cytometry*, **8**, 474.

MEADOWS, A.T. (1987). Bilateral anaplastic Wilms' tumors: change in ploidy following treatment. *Med. Pediatr. Oncol.*, **15**, 28.

MORAN, R.E., BLACK, M.M., ALPERT, L. & STRAUS, M.J. (1984). Correlation of cell-cycle kinetics, hormone receptors, histopathology, and nodal status in human breast cancer. *Cancer*, **54**, 1586.

RAINWATER, L.M., HOSAKA, Y., FARROW, G.M. & LIEBER, M.M. (1987). Well differentiated clear cell renal carcinoma: significance of nuclear deoxyribonucleic acid patterns studied by flow cytometry. *J. Urol.*, **137**, 15.

SANDSTAD, E. & HARTVEIT, F. (1987). Stromal metachromasia: a marker for areas of incipient invasion in ductal carcinoma of the breast. *Histopathology*, **11**, 73.

SCHMIDT, D., WIEDEMANN, B., KEIL, W., SPRENGER, E. & HARMS, D. (1986). Flow cytometric analysis of nephroblastomas and related neoplasms. *Cancer*, **58**, 2494.

TOOLE, B.P., KNUDSON, C.B., KNUDSON, W., GOLDBERG, R.L., CHI-RISS, G. & BISWAS, C. (1987). Hyaluronate-cell interactions in morphogenesis and tumourgenesis. In *Mesenchymal-Epithelial Interactions in Neural Development*, Wolff, J.R. (ed). Springer Verlag: Berlin.